# Sources of Lipopeptides and Their Applications in Food and Human Health: A Review

**DOI:** 10.3390/foods14020207

**Published:** 2025-01-10

**Authors:** Shuyi Chen, Sumin Chen, Xin Yu, Caijing Wan, Ying Wang, Lianxin Peng, Qiang Li

**Affiliations:** Key Laboratory of Coarse Cereal Processing, Ministry of Agriculture and Rural Affairs, Sichuan Engineering & Technology Research Center of Coarse Cereal Industrialization, School of Food and Biological Engineering, Chengdu University, Chengdu 610106, China; 18882882771@163.com (S.C.); chensumin76@163.com (S.C.); m18481822277@163.com (X.Y.); 13206009521@163.com (C.W.); 19938308064@163.com (Y.W.); penglianxin@cdu.edu.cn (L.P.)

**Keywords:** lipopeptide, food, medicine, antibacterial, synthesis

## Abstract

Lipopeptides (LPs) are widely sourced surface-active natural products with a wide range of functions and low toxicity, high potency, and good biodegradability. In this paper, we summarize, for the first time, the plant, animal, microbial, and synthetic sources of LPs. We also introduce the applications of LPs in food and human health, including (1) LPs can inhibit the growth of food microorganisms during production and preservation. They can also be added to food packaging materials for preservation and freshness during transportation, and can be used as additives to improve the taste of food. (2) LPs can provide amino acids and promote protein synthesis and cellular repair. Due to their broad-spectrum antimicrobial properties, they exhibit good anticancer effects and biological activities. This review summarizes, for the first time, the sources of LPs and their applications in food and human health, laying the foundation for the development and application of LPs.

## 1. Introduction

Lipopeptides (LPs) are important biological surfactants, which are compounds or complexes formed by linking lipids and amino acids through chemical bonds such as ester bonds or amide bonds. The structural differences in LPs are mainly reflected in the composition of the peptide chain, the length and type (linear or branched) of the fatty acid chain, and the type of chemical bond that connects the peptide chain with the fatty acid chain [1]. Based on these structural characteristics, LPs can be divided into two major categories: linear and cyclic. Most LPs belong to non-linear structures, which usually contain a large lactone ring. This ring is connected through the hydroxyl group of the C-terminal amino acid residue with other amino acid residues or fatty acid chains within the ring [2]. They are produced mainly by plants, microorganisms, and a small number of animals. Gram-positive *Bacillus* are the main source of lipopeptide production [3]. LPs may exist in natural foods or be generated during food processing, playing significant functions and fulfilling important roles [4]. Initially, it can impact the taste and consistency of foods, enhancing flavor. Research has indicated that yogurt containing LPs demonstrates improved strain stability and texture, increased EPS, reduced syneresis, extended shelf life, and enhanced flavor [5]. Furthermore, LPs are essential for regulating the stability and shelf life of food. Microbial spoilage poses a significant threat to food quality, leading to a considerable amount of food waste and impacting food safety [6]. With its antibacterial properties, LPs are widely used as antibacterial agents in food safety and treatment. The application of the supernatant on fruits and vegetables can effectively prolong the freshness period of food, and incorporating it into water can also help prevent the rapid deterioration of aquatic products [4,7]. Hybrid-designed membranes made of LPs and clay nanoparticles can be utilized in food packaging to inhibit microbial spoilage [8].

In addition, LPs also have several health effects and physiological functions. Due to their amphipathic characteristics, LPs are often used in cosmetics or care products. This can increase the emulsification, foaming, and moisturizing abilities of the products and reduce irritation [9]. Due to the abuse of antibiotics and the rise in bacterial resistance in the medical field, the need for the development of biostatic agents has become more pressing. The growth of bacteria, including those that are resistant to multiple drugs, can be significantly suppressed by LPs. The antibacterial effect is influenced by the length of the fatty acid chain and the peptide chain and is directly related to hydrophobicity. By modifying the structure and introducing modifying groups, the activity and stability of LPs can be increased [10]. LPs not only have antagonistic activity against fungi, bacteria, and Mycoplasma, but they also have significant antiviral activity against human immunodeficiency virus (HIV), herpes simplex virus (HSV), and foot-and-mouth disease virus [11].

The current research on LPs is still not perfect and has certain limitations. Some LPs can be challenging to extract from food matrices or may be prone to contamination and oxidation during the extraction process. They are sensitive to temperature, pH, microorganisms, and other factors during food processing and storage, necessitating high standards for the quality and stability of LPs. Cyclic LPs are primarily sourced from the secondary metabolites of microorganisms, resulting in a mixture of various types that can be challenging to differentiate. Due to their intricate structure and diverse biological activity, understanding the functional mechanism of these LPs presents a significant challenge [12].

Zhang examined the natural antibacterial LPs produced by *Bacillus* and their potential use in food preservation, while Pilz discussed the progress of LPs in the cosmetic and pharmaceutical industries [4,9]. Still, none of these studies have thoroughly detailed the different origins of LPs and their role in food and human health. This paper aims to provide an overview of the origins of LPs and the connections between LPs, food, and human health. By doing so, it offers valuable insights for the advancement of LPs and establishes a theoretical foundation for their utilization in the food industry and human health. Ultimately, this paper seeks to encourage the widespread application of LPs across various industries.

## 2. Lipopeptide Source

### 2.1. Plant Sources

Plant-derived peptides have been the focus of much attention due to their potential for a wide range of industrial and pharmaceutical applications. They can be isolated from various plants and their different organs. Extracted LPs, including defensins, thionins, heveins, and other varieties, are essential components that play important roles in nature [13,14]. In recent years, researchers have made significant progress in the extraction and study of plant lipids. Three LPs extracted from Camellia chinensis leaves were discovered to possess notable inhibitory effects on food spoilage bacteria and toxin-producing fungi, offering a fresh approach to food preservation [15]. Additionally, by utilizing liquid–liquid extraction and gas chromatographic separation on corn soaking water, scientists have discovered several amino acid-rich LPs that could be beneficial in the fields of agriculture and biotechnology [16]. A plant defensin peptide with antibacterial properties was successfully identified and characterized in the *Avena sativa* L. transcriptome. This peptide, which features eight conserved cysteine residues and four disulfide bridges, does not impact Gram sativa. It displays antibacterial activity against both positive and Gram-negative bacteria [17]. *Allium sativum* L., a widely used medicinal plant, was discovered to contain an antibacterial plant defensin peptide in its EST library. This peptide was effectively expressed using the Escherichia coli expression system, offering valuable information for the creation of new antibacterial agents [18]. The hydrolysate of pepper stalks was effectively converted into a raw material for iturin production through a combination of chemical and bacterial processes. This discovery offers innovative methods for eco-friendly manufacturing [19]. Astafieva’s research broadened the range of plant LPs by identifying three new antibacterial LPs from dandelion flowers. The study revealed that these peptides have unique cysteine-rich structures that are currently exclusive to dandelion flowers [20].

### 2.2. Animal Origin

Animal-derived LPs are primarily sourced from marine organisms, which are known for being high-quality suppliers of bioactive peptides. These peptides have shown antimicrobial, anticancer, immunomodulatory, and anti-inflammatory properties, making them highly valuable for various applications [21]. Three peptides identified in polar marine annelids displayed remarkable bactericidal activity under thermochemical conditions, despite differences in the C-terminal parts of their core peptides in terms of amino acids and structures [22]. The liver of *Lethenteron camtschaticum* synthesizes peptides with antibacterial properties that are crucial for the innate immune system of organisms and are extremely valuable in boosting immunity against pathogens [23]. Novel peptides in grass carp interferon with effective therapeutic efficacy were identified in two models: clinically severe extraintestinal pathogenic Escherichia coli and mouse endotoxemia [24]. Freshwater crayfish are also an important source of biologically active compounds, with peptides being produced in blood cells and stored in granules. These peptides are released in response to stimuli [25]. Anti-lipopolysaccharride factor (ALF), an antimicrobially active peptide originally isolated from *Limulus polyphemus* and extracted from South American *Litopenaeus vannamei*, activates sequence-independent innate antiviral immune responses, thereby enhancing protection against WSSV infection [26]. LPs from other animal sources are quite scarce. The peptides found in wasp venom serve different purposes, including degranulation activity and chemotactic properties. Some of these peptides exhibit antibacterial and immunomodulatory activities [27]. The whey peptide mixture from buffalo colostrum was found to have a wide range of activity against both Gram-positive and Gram-negative pathogens in a proteomic analysis [28]. Scorpion venom contains a high concentration of antibacterial peptides, which have varying levels of hemolytic and antibacterial properties when tested in a lab setting. These peptides show promise as potential therapeutic medications [29]. Peptides with antioxidant activity can be extracted from bovine and goat milk through proteolytic solid-state fermentation of *Aspergillus oryzae* and *Aspergillus flavipes* [30].

### 2.3. Microbial Sources

#### 2.3.1. Bacterial Sources

LPs can be produced by a variety of microorganisms, such as bacteria, fungi, and algae. Among these, *Bacillus* spp. bacteria are the source of the vast majority of LPs [9]. The LPs produced by these bacteria exhibit a variety of biological functions due to differences in peptide chain length, amino acid types, and arrangements [31]. In industrial production, *Bacillus subtilis* is favored for its excellent performance in lipopeptide production (Table 1). Since 1957, scientists have been aware of the presence of LPs in microorganisms found in this soil. *Bacillus* subtilis, initially utilized as an antibacterial agent, has consistently shown its ability to produce various LPs in later research. Surfactin, specifically produced in these conditions, has emerged as a significant product in the realm of lipopeptide synthesis [4,32]. Apart from *Bacillus* bacteria, other types of bacteria, such as *Pseudomonas*, are also capable of producing LPs. *Pseudomonas*, for instance, can generate a variety of cyclic LPs, including sticky proteins and amphoteric proteins [33]. Additionally, *Streptomyces* strains can synthesize a diverse array of LPs that possess potent antibacterial effects through their interference with cell wall synthesis [34].

#### 2.3.2. Algae Sources

Algae have the ability to produce a wide range of bioactive compounds, including LPs that were extracted from methanol extracts of terrestrial blue-green algae [44]. The LPs obtained from seaweed are effective against various microbial pathogens, and the extraction techniques are user-friendly. They can be utilized for food preservation or incorporated into foods as functional ingredients, serving as potential sources of natural food preservatives [45]. Functional LPs were successfully extracted from *Himanthalia elongata* and *Palmaria palmata* to extend the shelf life of foods and produce edible films [46]. Through optimization of culture conditions and application of bioengineering techniques such as genetic engineering and metabolic engineering, the yield of LPs in cyanobacteria can be increased [47]. Light and microorganisms such as cyanobacteria and algae can also produce LPs with antibacterial effects [9]. The seaweed extracts (Padina sp.) and purified cyanobacteria (Ulva sp.) demonstrated antibacterial activity at concentrations ≤500 µg/mL against Gram-positive foodborne pathogens, including Listeria, *Bacillus* cereus, and *Staphylococcus aureus* [48]. The harsh environment of Antarctica leads algae to produce bioactive molecules necessary for their survival, cellular functions, and adaptation. These molecules, found in Antarctic algae, are rich in LPs with various properties such as antibacterial, anticancer, and antioxidant effects [49]. *C. humicola* is a diverse source of natural products and nutraceuticals containing bioactive compounds like carotenoids, alkaloids, fatty acids, and amino acids. These compounds exhibit strong pharmacological activities against microbial pathogens [50].

#### 2.3.3. Fungal Sources

The discovery of penicillin has made fungi an emerging source of bioactive secondary metabolites. Since 2000, 30 representative fungal genera have been found to produce LPs, which have enormous potential as drugs and biocontrol agents [51]. NZX, a novel derivative of the fungal antibacterial peptide plectasin, is the first fungal defensin peptide with confirmed activity against Streptococcus pneumoniae and methicillin-resistant *Staphylococcus aureus*. This non-toxic peptide can inhibit the clinical Mycobacterium tuberculosis strain and one multidrug-resistant (MDR) strain [52]. Cyclic and linear LPs with antimicrobial effects against phytopathogenic bacteria can be extracted from Aspergillus. Modification of the β-amino fatty acid chain of the cyclic LPs enhances the antifungal activity of the LPs [53]. *Trichoderma* secretes a variety of volatile compounds, including peptides, that are widely used in biological control, biological fertilizers, and industrial enzymes [54].

### 2.4. Synthetic Sources

LPs have a disadvantage in their high production cost and low yield. Since LPs are frequently mixed with other secondary metabolites, their separation and purification can be quite challenging, leading to a preference for artificial synthesis in industrial settings (Table 2). The construction of novel LPs through synthetic biology methods can improve the production of LPs in natural hosts and facilitate the development of their heterologous production to obtain a diverse range of natural biodiversity [43]. During the synthesis of LPs, different structural domains like adenylation and thiolation domains are involved in a series of enzymatic modifications and transport. Typically, it starts as a propeptide and is later transformed into a lipopeptide through enzymatic modification [55,56]. Artificially synthesized LPs are also highly active. Ma et al. successfully synthesized the anticancer lipopeptide P17, which has low cytotoxicity, low haemolysis, high serum stability and permeability, and can be self-assembled into spherical aggregates to encapsulate drugs [57].

## 3. Lipopeptide Foods

### 3.1. Food Functionality of LPs

**Figure 1:**
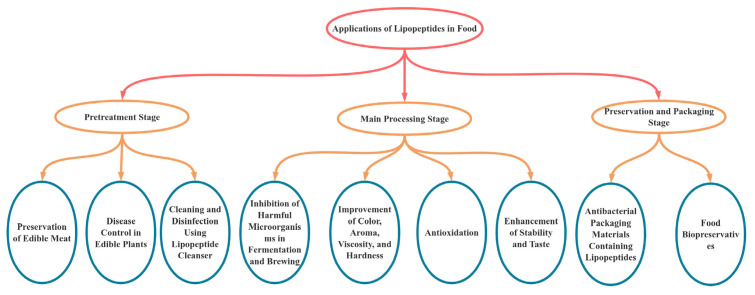
The role of lipopeptides in food.

LPs play multiple roles in food (Figure 1), especially bacteriocins isolated from the genus *Bacillus* spp. This bacteriocins have a wide range of antimicrobial functions against foodborne pathogens and can inhibit the microorganisms that cause food spoilage. Therefore, they are very promising biopreservatives. In the fermentation of natto, Qingguo sauce, and fermented bean curd with *Bacillus*, the lipopeptide demonstrated a strong antifungal effect that was notably more effective in inhibiting fungi compared to lactic acid bacteria [68]. In the prevention and control of edible plant diseases, LPs have a better inhibitory effect on Oryzae, are heat-stable, and are resistant to extreme acid and base. They also have a certain control effect on rice leaf blast. HZ-12 can inhibit apple rot caused by *Aspergillus niger*, and its antibacterial effect can reach 89.0%. This strain produces a lipopeptide with a strong antibacterial stability, and by genetically engineering a potent promoter, its antibacterial effectiveness can be enhanced even further [69,70]. The antifungal activity of iturin is related to its concentration. Inoculating iturin-rich supernatant into lemon and strawberry fruits can effectively prevent the expansion of fungal mycelia on diseased fruits and control their pathogenesis. Iturin also improves the resistance of loquat fruits to anthracnose [71]. The treatment of *Lupinus mutabilis* with LPs increased the chlorophyll and protein contents, as well as the enzyme activity of the seedlings. This had a positive effect on the physiological processes and overall growth of the seedlings [72]. Metals build up in water, minerals, rocks, and soil, where they are then taken in by plants through a combination of organic and inorganic molecules in various locations. By incorporating lipopeptide biosurfactants into a 2X CMC solution, vegetables can effectively repair and break down heavy metals like Cd and Pb [73].

The mechanism by which LPs extend the shelf life of food is primarily attributed to their unique biological activities and antibacterial properties. LPs substances can inhibit the growth and reproduction of harmful microorganisms in food, including common food contaminants such as *Escherichia coli* and *Salmonella enteritidis*. By damaging the cell membrane structure of microorganisms and altering their permeability, LPs cause the leakage of intracellular substances, thereby inhibiting the microbial life activities and extending the shelf life of food. In fruit and vegetable preservation, it can effectively inhibit food spoilage related strains such as *Aspergillus niger* and *Staphylococcus aureus*, inhibit fungal pathogens in fresh grapes and pear fruits after harvesting, and maintain the quality of fruits [74]. They also help maintain fruit quality and enhance the activities of SOD, CAT, POD, PPO, and PAL in tomato fruits. This demonstrates significant antifungal potential against *Alternaria Nees* in tomatoes [75]. High-cell-density cultivation enhances the secretion of LPs in Bacillus subtilis cultures, which better maintains the appearance quality and physiological indicators of grapes [76]. Lipopeptide-treated strawberries were able to extend their freshness by 3d [77]. In the wine industry, LPs can prevent grape wine products from being contaminated by fungi and the production of *ochratoxin* A (OTA), thereby maintaining flavor and taste and improving the stability of wine quality [78]. In meat products, LPs can inhibit the growth of Pseudomonas in catfish meat, reducing its toxicity and drug resistance, and playing a role in the preservation of fish meat [79]. In addition, feeding with LPs improved the growth performance, specific growth rate, weight gain, and survival rate of prawns. These tests revealed that the levels of digestive enzymes and immune enzymes in prawns fed LPs were also relatively high, suggesting that a lipopeptide diet can actively regulate digestion and immune activities of shrimp and increase disease resistance [80]. The cyclic peptide (BLS) produced by *Bacillus licheniformis* P40 has emulsifying properties with hydrophobic compounds and exhibits dual antibacterial and emulsifying activities in meat model systems, suggesting its potential as a food biopreservative or bio-detergent [81].

### 3.2. LPs and Food Packaging

Food packaging is an important part of ensuring the quality and safety of food during storage and transportation. The addition of ingredients with antibacterial effects can extend the shelf life of food, which holds great potential for the application of LPs in packaging. There are two main methods for incorporating LPs into packaging: (1) adding LPs to the polymer and (2) smearing the LPs on the surface of the material.

The nanocomposite membrane developed by combining lipopeptide and clay nanoparticles exhibited excellent mechanical strength, water resistance, and antibacterial properties [8]. In the field of nanofood packaging, the iturin component in the LPs not only successfully reduces the silver concentration in the material but also significantly enhances the antibacterial activity through efficient synthesis of AgNPs [82]. Polyvinyl alcohol (PVA)-based antibacterial films incorporating LPs and zinc oxide nanoparticles (ZnONPs) exhibit enhanced optical, thermal, mechanical, and water barrier properties. These films also demonstrate excellent antibacterial activity against biofilm-forming bacteria [83]. The LPs DCS1 are crucial in preserving the nutritional qualities of food by inhibiting the production of hydrogen peroxide and malondialdehyde (MDA) compounds. The emulsion’s nutritional value remained intact over the 23-day storage period, in contrast to the gel film. Mixing them directly into ground beef patties is more efficient than using coatings in preventing food oxidation and preserving food freshness [84]. The blend of *Bacillus* exopolymeric substance (EPS) and chitosan not only boosts the tensile strength of the film but also successfully prevents fungal contamination, extending the postharvest shelf life of fruits like mangoes while minimizing spoilage. Additionally, this composite film effectively slows down the rise in pH and total viable count (TVC) values, as well as lipid oxidation in fresh pork, guaranteeing the freshness and safety of meat products [85,86]. The PLC was used to encapsulate the peptide, forming a multilayer nanofiber mat. This achieved an encapsulation efficiency of up to 65% and excellent performance, with a maximum load of peptide per gram of PUL. This material can be used as part of active packaging [87].

LPs can be directly coated on the surface of food packaging materials due to their good emulsifying and antibacterial properties, as well as their non-cumulative toxicity to the human body. During the coating process of the film, the film can come into direct contact with another film, allowing the peptides to transfer and attach to the surface of the film through contact. Alternatively, the film can be immersed in a pre-prepared peptide solution, allowing the peptide molecules to naturally adsorb and cover the surface of the film [88]. In addition, the coating can also be accomplished through solvent casting. This means that the solution containing the peptides is evenly poured onto the film’s surface. As the solvent evaporates, the peptides are deposited and a protective coating is formed [89].

However, the application of LPs in food packaging also faces numerous problems and challenges. The production process of LPs is relatively complex, requiring high-precision technology and equipment support, which increases production difficulty and results in relatively high production costs [90]. In the food packaging industry, cost control is crucial, and therefore, the high cost of LPs may limit their widespread application in food packaging [73]. LPs also need to maintain sufficient stability to ensure they do not degrade or lose activity during the packaging process [91]. To ensure the effectiveness and safety of LPs in the packaging process, it is crucial to maintain their stability and prevent degradation or loss of activity. This means that appropriate measures must be taken to protect LPs during packaging to maintain their performance. Moreover, although LPs have the advantage of being biodegradable, which is environmentally friendly, caution must still be exercised in their use and disposal to ensure that they do not have a negative impact on the environment [92]. Therefore, when applying LPs in food packaging, it is necessary to consider their stability, biodegradability, and potential environmental impacts comprehensively in order to achieve a packaging solution that is both safe and environmentally friendly.

### 3.3. Lipopeptide Food Taste

Compared with synthetic surfactants, microorganism-derived surface-active compounds have properties such as low toxicity and biodegradability and have shown potential applications in many areas of the food industry [93]. LPs are natural, efficient, safe, and green. They not only optimize the taste of food, increase its richness, maintain the consistency and volume of baked goods, and emulsify fat globules to prevent them from aggregating, but they also affect the textural characteristics of baked goods and their sensory attributes, such as improvements in color, aroma, viscosity, and hardness [94,95].

Lipopeptide biosurfactants have the ability to stabilize and lower the surface tension between oil and water, enabling them to blend effectively and improve the textural properties of food, including hardness, chewiness, cohesiveness, adhesion, and firmness. This enhances the strength and flexibility of the food product [96]. For example, l-glutamic acid methyl ester in LPs can effectively increase the survival rate of fermenter cultures, resulting in enhanced flavor components and better stability of yogurt [5]. In culinary creations like salad dressings, breads, and cakes, LPs serve as emulsifiers that not only enhance product stability and flavor but also provide outstanding antioxidant, antibacterial, and antiadhesive properties [97]. By utilizing *Bacillus subtilis* FUA2155 and *Bacillus amylum* Fad WE starters in conjunction with white wheat flour, wheat bran, or buckwheat, along with the incorporation of 2.5–20% BWE into bread dough, one can successfully manage bread spoilage, enhance bread volume, and elevate the overall quality of bread [98]. In ice cream formulations, LPs are used as biosurfactants to enhance taste by improving creaminess, texture, and overall effects [99]. Moreover, LPs have shown considerable benefits in substituting conventional animal fats. When added as biosurfactants in regular biscuit recipes, they not only create a softer and more spongy texture without altering the product’s physical and physicochemical characteristics or energy content but also prove to be valuable in the biscuit industry. This highlights their potential in the creation of nutritious food options [100]. In probiotic products, such as yogurt, the addition of LPs not only significantly increases the survival rate of probiotics under stress conditions and ensures the stability of probiotic activity, but also further optimizes the flavor of the product by improving the taste and texture of yogurt [5]. In the production of biscuits, the biosurfactant *Bacillus* spb1 has proven to be a valuable addition. After the addition of this biosurfactant to the biscuit dough, the biscuits became softer, and the overall quality significantly improved [101]. These findings not only broaden the application range of LPs in the food industry, but they also provide new ideas and methods for food production to achieve higher-quality and healthier products.

## 4. LPs and Human Health

### 4.1. Antibacterial Effects

Some LPs have antioxidant and antibacterial properties and can help reduce tissue damage caused by oxidative stress and inflammation. Owing to their broad bactericidal spectrum, LPs have potential application value in medicine. Lipopeptide surfactants exhibit low toxicity towards human cells and possess strong biocompatibility. They are able to shield tissues from oxidative harm, enhance blood clotting and skin wound healing, as well as reduce inflammation and pain resulting from microbial growth [102].

**Figure 2:**
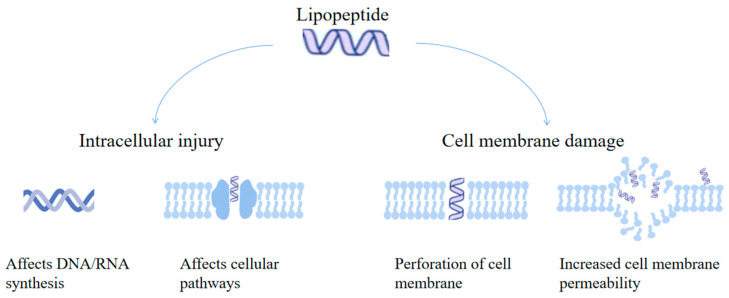
Bacteriostatic mechanism of lipopeptides. Lipopeptides prevent bacterial growth by either interacting with components in the envelope or by causing damage to the cell membrane.

LPs have three main antibacterial mechanisms (Figure 2). (1) Interactions with intracellular substances: LPs can act on intracellular targets and interact with substances such as DNA to exert their antibacterial effects. For example, the LPs produced by *Bacillus subtilis* inhibit the gene expression of *Salmonella typhimurium* by increasing the mRNA levels of intestinal barrier-related genes in damaged intestinal tissue [103]. LPs can affect multiple pathways of *L. monocytogenes*, including peptidoglycan biosynthesis, membrane transport, cell metabolism, ATP synthesis, and stress response activation, thereby affecting the gene expression of this bacterium and inhibiting its growth [104]. (2) Cell membrane perforation: a significant property of LPs is their ability to perforate biomembranes. They act directly on the biofilms of bacteria and fungi, forming pores in the membranes and causing an imbalance in permeability, which leads to cell death. By replacing rhamnose with amino acids in rhamnolipid, 43 LPs were successfully produced. These LPs can effectively inhibit the biofilm formation of *Candida albicans* by preventing the adhesion of this bacterium [105]. Coryxin, a novel biosurfactant produced by the *Corynebacterium xerosis* NS5 strain, exhibits inhibitory and destructive effects on biofilm formation and displays antibacterial activity against both Gram-positive and Gram-negative bacteria [106]. In addition, the binding of the cationic lipopeptide gramibactin to lipoteichoic acid can also destroy the plasma membrane of *Staphylococcus aureus* [107]. Pumilacidin, isolated from *Bacillus subtilis* NITDID1, can successfully prevent the initial deposition of microbial biofilms on silica gel surfaces and significantly reduce the formation of biofilms [108]. (3) Inducing cell death by increasing cell membrane permeability: LPs can be effectively inserted into lipid membranes. As the concentration of lipopeptide increases, it can continuously increase the zeta potential of the membrane surface, thereby potently damaging lipid membranes. Changes in the permeability of the cell membrane eventually lead to cell death. Studies have shown that the overall positive charge of LPs can lead to poor hemolysis and toxicity in normal living cells, as well as rapid clearance from the blood circulation. Self-assembly into negatively charged spherical nanostructures relieves protein adsorption and prolongs blood circulation in the body for the removal of bacteria from the body [109].

### 4.2. Bioactivity and Immunomodulation

**Figure 3:**
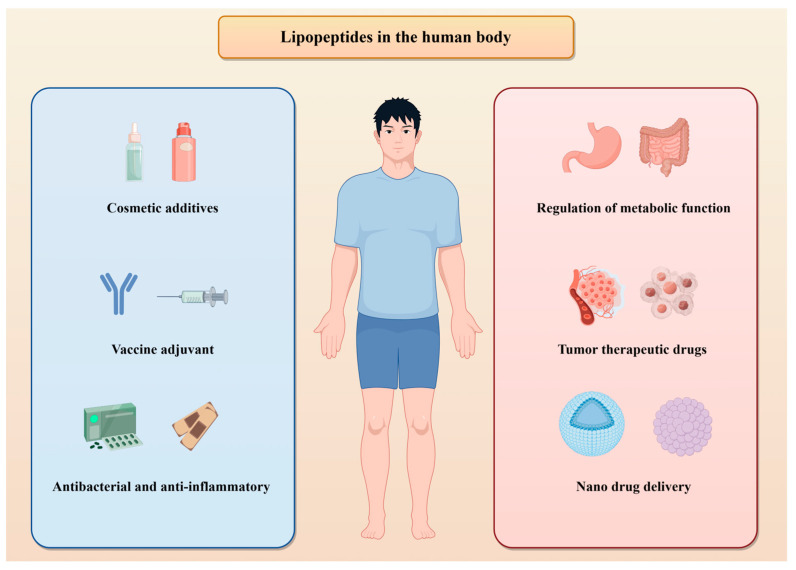
The role of lipopeptides in human health.

Due to their unique structural features, including multiple hydrogen bond interactions and lipophilicity, lipopeptide drugs can enhance their affinity for biomembranes, leading to improved targeting and insertion into tumor cells (Figure 3). This allows them to specifically target tumor cells and induce cell death by disrupting the biofilm system or damaging mitochondrial membranes. To enhance the effectiveness of lipopeptide as a drug in tumor treatment, it can be combined with nonionic three-block copolymers (polyamides). This combination not only boosts its antibacterial properties but also reduces hemolytic side effects, making it more harmful to tumor cells and increasing its toxicity significantly [110]. By utilizing a homing peptide (NGR) as the targeting ligand attached to liposomes, the surface was able to effectively target CD13 receptor-positive cancer epithelial cells and angiogenic endothelial cells. This resulted in receptor-mediated uptake and apoptosis-induced cell death in certain cells, showcasing promising therapeutic implications for treating aggressive pancreatic cancer [111]. Self-assembling lipopeptide nanotherapeutic drugs contain two linoleic acid molecules and exhibit excellent antitumor activity and antibacterial function against both sensitive and drug-resistant strains. Increasing the amount of hydrophobic linoleic acid in a lipopeptide is a good way to improve efficacy in both tumor cells and bacteria [112]. Bacitracin D, produced by the *Bacillus amyloidus* strain Fiply 3A, showed selective toxicity against three different cancer cell lines. This lipopeptide selectively induces ROS-mediated DNA damage, which results in the release of cytochrome C from mitochondria and ultimately causes apoptosis in cancer cells [113]. A new class of lipid-peptide clusters, isolated and identified from marine algal bacteria, exhibited significant changes in the length of the fatty acid chain and amino acid substitutions in the peptide chain. Cell viability analysis revealed that the LPs had good anticancer activity against cancer cell lines. These compounds do not exhibit toxicity against the noncancerous lung fibroblast line MRC5 and have good potential for application [114]. Synthesis of an amphiphilic cationic P17 lipopeptide with an α-helical structure, low cytotoxicity, low hemolysis, high serum stability, and membrane-penetrating ability was achieved through solid-phase synthesis. This lipopeptide can self-assemble into spherical aggregates, which can then encapsulate anticancer drugs to form nanomedicines and achieve combined therapeutic effects [57]. In addition, dendritic lipopeptide (DLP)-modified multilevel targeting liposomes (Mtlips) technology has shown great potential in drug delivery. This innovative delivery system is integrated into the hydrogel matrix and can penetrate the dense stratum corneum to reach the epidermis where melanoma is located. Through high permeability, it significantly enhances the payload of drugs in tumor tissues and selectively accumulates in mitochondria, thereby increasing drug toxicity. This local and sustained release method provides new possibilities for tumor treatment [115].

In the field of vaccine research and development, LPs have attracted much attention because of their unique properties, which are both antigens and adjuvants. The design of lipopeptide antigens is based on the accurate sequence of the antigenic protein, which can precisely stimulate the receptors on the cell surface to elicit a specific immune response. A great advantage of peptides is that they are easy to design and can be efficiently prepared via automated synthesis methods. In addition, peptides can also be combined with lipids and other molecules and used as adjuvants or antigen presentation tools in subunit vaccines to further increase their efficacy [116]. Liposomes are vaccine adjuvants that allow for the co-encapsulation of antigens and immunostimulatory agents. The novel geminic lipid peptide (AG2-C16), which utilizes CpG-ODNs as immunostimulatory agents, can activate the Th2/Th17 spectrum and induce a strong TH1 response [117]. When the wheat storage protein gliadin is used as an antigen, the lipopeptide PCSK can activate the TH1 subgroup and enhance mucosal and systemic immune responses [118]. Cationic liposomes (DDAAs) have been used to encapsulate lipopeptide-based GAS vaccine candidates through the film hydration method. Intranasal administration can induce mucosal and systemic immunity in mice, indicating a very promising delivery route for intranasal vaccines [119]. Brar et al. showed that the water-soluble TLR2 agonistic lipopeptide Pam enhanced the immunogenicity of recombinant SARS-CoV2 and hepatitis B antigen in mice, making it a promising TLR2-targeting adjuvant [120].

### 4.3. Nutrition and Metabolism

Lipopeptide, a byproduct of protein breakdown, serves as a source of crucial amino acids for the body, aiding in protein synthesis, cell repair, and overall maintenance of normal nutritional status and metabolic function. Its distinctive biological activity makes it a popular ingredient in cosmetics and health products [9]. As skin ages, there is a simultaneous occurrence of skin cell apoptosis and a reduction in elasticity. When specific peptides work together, they can enhance the regenerative capacity of skin cells in vitro through complementary and synergistic mechanisms [121]. Studies have shown that lipopeptide products can restore effective cell metabolism, significantly improve signs of skin aging, and can be used as anti-aging solutions [122]. LPs have free radical scavenging activity and can replace ceramide to improve skin surface roughness and help skin barrier formation [123]. In traditional cosmetic production, amino acids, vitamins, antioxidants, and other ingredients are added to the formula at each production stage as separate raw materials. However, lipopeptide is a natural mixture of ingredients that offer certain advantages in production [124]. Owing to the amphipathic characteristics of lipopeptide, it has good emulsifying and foaming properties. Therefore, it can be used in shampoos, shower gels, and detergents, reducing skin damage and promoting biodegradability [125].

In the regulation and maintenance of metabolic function in the human body, LPs also play various roles. Surfactin, a natural lipopeptide, prolongs insulin fibril formation, improves insulin unfolding, retains the α-helix content, and inhibits the upregulation of proinflammatory genes induced by insulin amyloid. It can be used as an additive in biopharmaceutical preparations to enhance the stability of insulin [126]. The lipopeptide is composed of the artificial oligomeric amino acid succinyl-tetraethylenepentamine (Stp) and various amino acid trimers. Both of these components can effectively aggregate pDNA with low cytotoxicity and deliver nucleic acids to retinal cells for eye treatment [127]. There is a close relationship between the bioactive molecules extracted from *Bacillus* and the molecular mechanisms of probiotics. In the intestinal environment, especially in the small intestine, the presence of oxygen and nitrate may lead to the dominant growth of facultative anaerobic bacteria. These bacteria control the respiratory electron acceptors in the intestine. However, clonal *Bacillus* spp. secrete active molecules that can influence and promote the growth of the intestinal flora. This helps shape and maintain a healthy composition of the gut microbiota [128].

### 4.4. Lipopeptides Toxicity

The toxicity of LPs primarily depends on their specific types, structures, and conditions of action, as different types of LPs may exhibit different toxic characteristics. The H6 lipopeptide surfactant isolated from *Pseudomonas* bacteria has been proven to effectively inhibit the growth of Saprolegnia [129]. Korbut et al. tested the toxicity of H6 and found that it exhibited low to moderate toxicity to free-living organisms at different trophic levels. Among them, *Daphnia magna* was the most sensitive, with a median lethal concentration (LC50) of 20 mg/L, zebrafish embryos were affected at a concentration of 27 mg/L, while green algae showed higher tolerance and were only inhibited at a concentration of 170 mg/L [130]. Additionally, Ben Ayed and others assessed the potential in vivo toxicity of the A21 lipopeptide by administering single doses ranging from 75 mg to 1000 mg/kg body weight (bw) to mice and observing them for 28 days. The results showed that A21 had no significant impact on mouse body weight or hematological parameters, indicating that it may be a promising product [131]. Similarly, toxicity studies of the SPB1 lipopeptide in mice also showed that daily intake at doses below 47.5 mg/kg bw had no significant adverse effects on hematological parameters and serum biochemical data. This lipopeptide also has the potential to prevent thrombosis [132]. Abdille et al. evaluated the toxicity of Drs B2 in mice and found that a 14-day subacute toxicity test did not cause significant changes in biochemical, hematological, and histological parameters. However, in a 90-day subchronic toxicity test, inflammatory responses in the spleen were observed [133]. Similarly, peptide P34 also caused similar effects in mice after 21 days of administration [134]. Wu et al. examined the subacute toxicity of S-thanatin by continuously intravenously injecting S-thanatin into ICR mice. The results showed that S-thanatin had no effect on hematological parameters and was a safe peptide for preclinical trials [135]. Furthermore, studies by Ba et al. have shown that phosphorylation can reduce the toxicity of peptides and increase their stability, thereby prolonging the survival time of animals infected with bacteria [136]. In summary, many LPs are safe under appropriate conditions. These toxicity studies not only provide important support for further application of LPs in food and medicine, but also offer valuable references for future research and development.

## 5. Conclusions and Outlook

Under the rapid development of biotechnology and food engineering, significant progress has been made in the research of LPs. This paper summarizes the four major sources of LPs, including microorganisms, plants, animals, and chemical or biosynthetic methods. Additionally, it elaborates on the important roles of LPs in the food industry and human health. This will lay the foundation for the use of LPs in promoting human health and encouraging their application in food. Furthermore, it will help establish a better understanding of the relationship between LPs and human health, leading to more extensive applications.

Currently, the complex structure and low yield of LPs limit their large-scale production, thereby constraining their widespread application. This also poses challenges for the transition of LPs from laboratory research to clinical application. In the future, we can continue to explore more diversified sources of lipopeptides, such as using genetic engineering to modify microorganisms to increase the yield and specificity of LPs or searching for new microbial resources in extreme environments to develop LPs with unique functions. Secondly, as bioactive molecules, the potential of LPs in the prevention and treatment of chronic diseases should not be ignored. For chronic diseases such as cardiovascular diseases, tumors, and neurodegenerative diseases, strengthening the correlation between LPs and these diseases will provide a new perspective for disease management. With more in-depth studies on the relationship between the structure and activity of LPs, we will be able to reveal more intrinsic mechanisms of lipopeptide activity, including its effects on food processing and metabolic pathways in the human body. This not only helps us better understand the action patterns of LPs in food, but also further expands their application prospects for human health.

## Figures and Tables

**Table 1:** Different LPs of *Bacillus* origin.

Type	Source	Role	References
Surfactin	*B. subtilis*, *B. amyloquefaciens*, *B. velezensis*, *B. brevis*	It can reduce surface tension and form micelles and is used in the detergent and emulsifier industries. It has an effective antibacterial effect on various microorganisms.	[35]
Iturin	*B. subtilis* *B.velezensis*, *B. amyloquefaciens*	Destroys cell membranes by interacting with the lipid components of fungi and bacteria, resulting in cell lysis. It also stimulates plant growth by improving nutrient uptake, protecting plants from pathogens, and promoting root system development.	[36]
Fengycin	*B. subtilis*, *B. amyloliquefaciens*, *B. thuringiensis*	It has effective antifungal activity against plant pathogens and filamentous fungi.	[37]
Kurstakins	*B. licheniformis*, *B. mojavensis*	It has an antibacterial effect against foodborne pathogens.	[38,39]
Bacillomycin	*B. amyloliquefaciens*, *B. velezensis*	It has an inhibitory effect on a variety of plant pathogens.	[40,41]
Polymyxins	*Paenibacillus polymyxa*	Inhibits most Gram-negative bacteria.	[42]
Licheniformin	*B. licheniformis*	It has antagonistic effects against Staphylococcus and yeast-like bacteria.	[43]

**Table 2:** Methods and uses of artificially synthesized LPs.

Substrate	Methods	Use	References
Silk peptide	Grafting of fatty acid hydrophobic chains onto silk peptides for N-acylation.	Endows the lipopeptide with improved emulsification performance.	[58]
Cyclolipeptide	Metal ion-induced cyclolipopeptide self-assembly and reconstruction into amphipathic particles.	Complex LPs are prone to coming into contact with and destroying microbial membranes.	[59]
IsocyanoLPs (INLPs)	“Isocyanosynthase” or nonheme iron(II) and α-ketoglutarate (KG)-dependent dioxygenase synthesis.	As potential drug targets for TB treatment.	[60]
Starch	Amylase is produced by *Pichia pastoris* to decompose starch in food waste.	Significantly increased bioconversion was observed from food waste to the production of LPs.	[61]
Short peptide	Two identical hydrophilic amino acids, X, and several kinds of glycine form 2–4 peptides. Palmitic acid is modified at the N-terminus and amino modification at the C-terminus.	Enhancement of the antibacterial effect of LPs and their activity against bacterial mastitis in mice.	[62]
Myxococcus xanthus	Type IIS endonucleases and synthetic DNA platforms.	For the production of various heterologous LPs.	[63]
Lipopeptide	The peptide moiety is functionalized with thiol-responsive groups, incorporated into liposomes, and reacts with the thiol-bearing peptide epitopes.	Used in the preparation of liposomal vaccines.	[64]
Surfactant	The CuSO_4_-5H_2_O solution was mixed with a biosurfactant solution and hydrazine hydrate was added.	As potential candidates for antimicrobial, antioxidant, anticancer and antidiabetic activities.	[65]
Iturin	Synthesis of silver nanoparticles (Ag-NPs) using nanotechnology.	For the control of Fusarium crown rot in wheat seedlings.	[66]
KLA and RGD peptides	Solid-phase synthesis.	As an excellent drug carrier, it has a combined anticancer effect.	[57]
Iturin	Natural chemical ligation (NCL) was used to achieve the synthesis of the parent peptide macrocycle, and the lipid moieties were then linked via the CLipPA technique using regenerated free thiols.	Altered biological and physical properties.	[67]

## Data Availability

No new data were created or analyzed in this study. Data sharing is not applicable to this article.
